# Role of neutrophils in cutaneous lupus erythematosus

**DOI:** 10.1111/1346-8138.17036

**Published:** 2023-11-27

**Authors:** Toshiyuki Yamamoto

**Affiliations:** ^1^ Department of Dermatology Fukushima Medical University Fukushima Japan

**Keywords:** lupus erythematosus, NETs, plasmacytoid dendritic cell, type I interferon

## Abstract

There are various types of cutaneous lupus erythematosus (CLE), either with or without the association of systemic lupus erythematosus (SLE). In some of the subtypes of cutaneous lupus, histopathology reveals neutrophil infiltration in the lesional skin; however, the significance of neutrophils in CLE is not yet fully elucidated. Recent studies have shown that neutrophil extracellular traps (NETs) formation by activated neutrophils is observed in several types of CLE, including lupus panniculitis, subacute lupus erythematosus, and acute lupus erythematosus, although the number of reports is small. Excessive NETosis, due to either increased NETs formation or defective clearance of NETs, may play a role in the induction of autoimmunity and autoantibody production in SLE, as well as endothelial damage, thrombus formation, and vascular damage in the lesional skin. CLE is an excessive interferon‐driven autoimmune disease. Plasmacytoid dendritic cells are located in lupus erythematosus skin and contribute to the etiology of skin lesions as a main producing cell of type I interferon. Neutrophils, monocytes, and keratinocytes also produce type I interferon via several triggers. Neutrophils play an important role in the innate immune response in SLE. In this review, several types of CLE with neutrophil infiltration, as well as the role of neutrophils are discussed.

## 1 INTRODUCTION

Cutaneous lupus erythematosus (CLE) can be observed in around 70%–80% of patients with systemic lupus erythematosus (SLE).^1^, ^2^ CLE lesions are triggered by several mechanisms, such as epidermal damage by ultraviolet light, leading to activation of an innate immune response, overproduction of inflammatory cytokines, vasculitis/vasculopathy, autoimmunity, production of autoantibodies against nuclear antigens, and immune complexes. Among these aspects, SLE is characterized by dysregulation of type I interferon (IFN) signaling. Plasmacytoid dendritic cells (pDCs) are triggered to produce type I IFN in response to apoptotic cells and neutrophil NETs; pDCs accumulate in the lesional skin of CLE.^3^ pDCs located in the dermo‐epidermal junction express granzyme B and colocalize with perforin‐expressing cytotoxic T cells, which suggest that pDCs may play a more direct role in the induction of apoptosis in CLE skin.^4^

Systemic lupus erythematosus has aspects of both autoimmune and autoinflammatory diseases.^5^ The innate immune system initiates abnormal autoimmunity, and type I IFN is one of the key molecules in innate immunity and SLE.^6^, ^7^ The presence of activated neutrophilic infiltrate in early and evolving lesions of CLE is a well‐known histopathological feature. In particular, neutrophils play an important role in some types of CLE, such as neutrophilic dermatosis and bullous lupus erythematosus (LE). Ultraviolet light‐induced damage of keratinocytes generates type I IFN, such as IFN‐κ, and releases cellular debris from apoptotic keratinocytes, which increase NETs formation as well as the production of autoantibodies. In this review, the roles of neutrophils, type I IFN, and pDCs in the pathogenesis of CLE lesions are examined, and local NETs formation in several types of CLE is demonstrated.

## 2 ROLE OF NEUTROPHILS IN LUPUS ERYTHEMATOSUS

Neutrophils play an important role as drivers of immune dysregulation in autoimmune diseases, including SLE.^8^ Several studies have suggested the role of aberrant neutrophil functions and their relevance to lupus pathogenesis.^9^ Neutrophil granulocytes are early responders in the course of tissue damage. Neutrophils produce antimicrobial peptides and reactive oxygen species and form NETs consisting of chromatin, histones, and DNA‐attached granular proteins. Neutrophils are primed to undergo NETs formation in the skin of patients with SLE. Immature, low‐density granulocytes (LDGs) are a proinflammatory subset of neutrophils that were initially described in SLE. LDGs are increased in number in the peripheral blood of patients with SLE. LDGs in SLE have an enhanced capacity to undergo cell death through the formation of NETs. LDGs in SLE enhance production of type I IFN and mitochondrial reactive oxygen species, which promotes mitochondrial DNA oxidation and the dysregulation of NETs formation.^10^ Lupus neutrophils are primed to make more amounts of NETs than neutrophils from healthy controls. Neutrophils from healthy controls generate more NETs when they are exposed to lupus plasma compared with plasma from healthy controls.

Increased NETs formation results from an imbalance between increased production and reduced degradation of NETs. SLE patients with a reduced ability to remove NETs have lower serum complement levels.^11^ Defective clearance of NETs or the remnants results in long‐term exposure to nuclear and cytoplasmic autoantigens, leading to chronic inflammation and autoimmune diseases. Furthermore, the release of NETs leads to pDC activation through endosomal toll‐like receptors (TLRs), with upregulation of type I IFN.^12^, ^13^

Neutrophil infiltration is observed in some of the subtypes of CLE, including acute LE, discoid LE, and lupus panniculitis. For example, patients with bullous LE frequently develop lupus nephritis, suggesting that activated neutrophils cause skin and kidney inflammation. Neutrophils activate both T cells and B cells. NETs can prime T cells to produce inflammatory cytokines and induce B cells to promote autoantibody production. Neutrophils may function as the messenger of injury signals (i.e., ultraviolet light) and migrate from the sun‐exposed skin to the kidney and induce nephritis.^14^

## 3 NEUTROPHIL INFILTRATION IN CUTANEOUS LUPUS ERYTHEMATOSUS

### 3.1 Neutrophilic urticarial dermatosis

Neutrophilic urticarial dermatosis presents with erythematous papules and plaques, urticarial‐like erythema, or annular erythema.^15^, ^16^, ^17^, ^18^ Histopathology reveals interface changes with vacuolar or liquefaction degeneration, perivascular and interstitial neutrophil infiltration below the epidermis and upper dermis with nuclear debris, and extravasation of red blood cells. Dermal mucin deposition is sometimes observed. Previously, those lesions were termed non‐bullous neutrophilic dermatosis; however, recently, several terms have been proposed such as neutrophilic urticarial dermatosis, SLE‐associated neutrophilic dermatosis, autoimmunity‐related neutrophilic dermatosis, and neutrophilic skin lesions in autoimmune connective tissue diseases. Although Sweet syndrome‐like dermal neutrophilic infiltrations are observed, the presence of interface dermatitis distinguishes SLE‐associated neutrophilic urticarial dermatosis from Sweet syndrome.

### 3.2 Bullous lupus erythematosus

Tense vesicles, blisters, and bullae appear on the sun‐exposed areas, as well as non‐sun‐exposed regions such as the trunk and extremities. Histopathology reveals subepidermal edema and bulla, neutrophil infiltration, neutrophilic microabscesses, and nuclear dust in the papillary dermis. Mucin deposition is apparent in the reticular dermis.^19^ Bullous LE is related to autoantibodies to type VII collagen, thus differentiation from epidermolysis bullosa acquisita is sometimes difficult.

### 3.3 Lupus erythematosus profundus

Lupus erythematosus profundus (LEP) presents with subcutaneous nodules predominantly involving the face, upper arm, back, waist, and thigh. Rarely, ulceration can be observed.^20^ Although the triggering causes of ulceration in LEP are uncertain, microangiopathic processes, such as segmental fibrinoid vascular necrosis, small vessel thrombosis, necrobiotic changes in the subcutaneous tissues caused by vascular changes, and dense angiocentric lymphocyte infiltrates, have been proposed. Recent studies have suggested that pDCs and type I INF play an important role in the pathogenesis of LE, and CD123 immunostaining may be helpful to differentiate LE from cutaneous T‐cell lymphoma.^21^, ^22^ pDCs secret proinflammatory cytokines, produce chemokines, and express costimulatory molecules, all of which contribute to the pathogenesis of LE.

### 3.4 Urticarial vasculitis

Urticarial vasculitis is a small vessel vasculitis which clinically presents with urticarial erythema, annular erythema, sometimes with purpura on the trunk and extremities. Histopathology reveals leukocytoclastic vasculitis in the upper dermis with interstitial neutrophil infiltration and nuclear debris. Patients often have SLE, Sjögren syndrome, and rheumatoid arthritis. Both hypocomplementemia and normocomplementemia are associated.

### 3.5 Amicrobial pustulosis of the folds

Amicrobial pustulosis of the folds (APF) mainly affects the scalp and ear canals as well as intertriginous areas such as the axillary, groin, and perianal regions. APF is characterized by sterile small pustules and erythemas. Histopathologically, subcorneal or intraepidermal neutrophil infiltration sometimes forms spongiform pustules, parakeratosis, and cellular infiltrates in the upper dermis. APF is a rare condition and is included as one of the neutrophilic dermatoses. APF is known to occur in patients with autoimmune diseases such as SLE. Marzano et al. proposed diagnostic criteria for APF that require association with one or more autoimmune disorders, positive antinuclear antibody at more than 1:160, and the presence of one or more serum autoantibodies.^23^ In addition, APF has recently been suggested to be on the spectrum of autoinflammatory disorders, and several cases of APF in association with neutrophilic dermatosis, such as palmoplantar pustulosis and pyoderma gangrenosum, have been reported.^24^, ^25^

## 4 NETS FORMATION IN CUTANEOUS LUPUS

Neutrophils play an important role in the initiation, promotion, and perpetuation of immune dysregulation via proinflammatory cytokine production, tissue damage through reactive oxygen species, and the formation of NETs.^26^ NETs are composed of extracellular structures such as DNA, histones, and neutrophil granules containing neutrophil elastase, and myeloperoxidase.^27^ Recent studies have shown increased NETs expression in the skin tissues of LE.^28^, ^29^, ^30^

**FIGURE 1:**
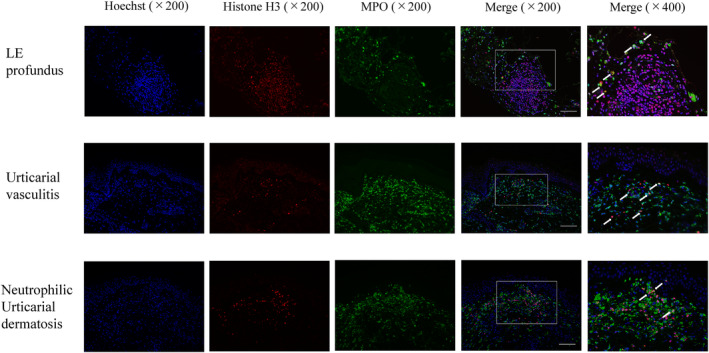
Biopsy specimens taken from the lesional skin of lupus erythematosus profundus, urticarial vasculitis, and neutrophilic urticarial dermatosis, and were stained with Hoechst, Histone H3, and myeloperoxidase. Merged photos show the presence of local NETing cells (yellow‐colored cells) in the upper dermis and subcutis (arrows). (Scale bars; 100 μm).

Safi et al. compared the amounts of neutrophils producing NETs in CLE between different subtypes.^30^ To detect local expression of NETs, DNA was immunofluorescently stained with Hoechst 33342, myeloperoxidase, and citrullinated histone H3 (citH3). Merged photos showed the presence of NETs in the lesional skin of CLE, including LE profundus, urticarial vasculitis, and neutrophilic urticarial dermatosis (Figure 1), whereas NETs were not observed in normal skins. These results suggest that NETing neutrophils play a significant role in the development of some CLE, accompanied by neutrophil infiltration.

NETs promote inflammation and skin injury; thus, excessive levels of NETs can damage the epidermis and vessels. NETs regulate inflammatory cytokines in response to alarmins. NETs stimulate macrophages to secrete interleukin (IL)‐1β and recruit neutrophils, thereby further promoting their own progression.^31^ Moreover, NETs can stimulate airway epithelial cells to secrete IL‐1 family cytokines such as IL‐36. Neutrophil elastase cleaves IL‐36 receptor antagonist into its highly active form.^32^ In addition, neutrophil‐derived proteases escalate inflammation by IL‐36‐α, −β, and ‐γ maturation and activation.^33^ The positive feedback loop between neutrophils and NETs promotes further formation of NETs, which act on keratinocytes. NETs may play a pathogenic role in some of the types of CLE by amplifying inflammation and tissue damage.

Neutrophils infiltrating the skin and kidney release IL‐33‐bearing NETs.^34^ Lupus NETting neutrophils are prone to producing IL‐33 alarmin, which induce a robust IFN‐α response by pDCs through the ST2L receptor. Abnormal NETosis may be caused not only by increased NETs formation but also reduced degradation of NETs.^35^ Patients with active SLE with hypocomplementemia have a reduced ability to remove NETs.^11^

## 5 TYPE I INF

Immune complexes formed by autoantibodies and endogenous nucleic acids are supposed to result in pDC stimulation. pDC is the main source of IFN‐α. Conversely, type I IFN is suggested to drive maturation of pDCs.^36^ In addition, neutrophils are also an important source of type I IFN. Monocytes are also a source of type I IFN after UV‐triggered injury in mice skin, and follicular dendritic cells produce IFN‐α in response to self‐immune complexes. Keratinocytes are also reported to produce a robust type I IFN, especially IFN‐κ.^37^

B‐cells have been implicated in the pathogenesis of SLE, due to the production of autoantibodies and cytokines. B‐cells are reported to infiltrate the lesional skin of discoid LE,^38^, ^39^ and high expression of B‐cell activating factor (BAFF) is observed in CLE including LE profundus.^40^ IFN‐α is a key cytokine in the pathogenesis of SLE and has multiple effects, one of which is upregulation of BAFF.^40^ Recent studies have shown that patients with chronic CLE share B‐cell abnormalities and expansion of effector B‐cell subsets, suggesting that chronic CLE may benefit from B‐cell targeting therapies.^41^ In particular, CD20‐, CD27 (memory B‐cells)‐, and CD79a‐positive B‐cells were observed in the subcutis, suggesting the role of B‐cells in LE profundus.

Plasmacytoid dendritic cells robustly secrete type I IFN in response to TLR 7/8 and TLR9 stimulation.^42^ In addition, pDCs secrete cytokines such as IL‐6 and tumor necrosis factor‐α, and control the expression of many inflammatory cytokines. Activated pDCs lead to the production of type I IFN, and persistent activation of type I IFN plays a key role in the pathogenesis of SLE, via lymphocyte attraction and expansion, as well as B‐cell hyperactivity via BAFF.^43^

## 6 PDC‐TARGETED AND IFN‐TARGETED THERAPY

As described above, pDCs are reported to play an important role in CLE. pDC‐targeting therapy by a monoclonal antibody against BDCA2, a pDC‐specific receptor involved in type I IFN production, has been shown to be beneficial to CLE. In a phase 2 trial, litifilimab, a humanized monoclonal antibody against BDCA‐2, significantly improved the CLE Disease Area and Severity Index‐activity score in 26 patients (50 mg), 25 patients (150 mg), and 48 patients (450 mg), over a period of 16 weeks.^44^

Type I IFN upregulates cytokines, chemokines, and adhesion molecules which recruit and activate pDCs as well as other inflammatory cells. The initial trigger induces synthesis of small amounts of type I IFN, releasing cellular debris from apoptotic cells. IFN represents a link between innate and adaptive immune responses. Hydroxychloroquine (HCQ) inhibits the production of IFN‐α by pDCs stimulated with TLR7 and TLR9 agonists, and is widely used for SLE and CLE. HCQ inhibits the production of various proinflammatory cytokines and prevents endosomal TLR activation and type I IFN production by pDCs.^41^ Chemokine receptor type 4 (CXCR4) is a potential regulator of pDC activation. A small molecule ligand of CXCR4 was shown to prevent nephritis in a lupus murine model, and decrease IFN‐α secretion in human SLE patients.^45^ Anifrolumab is an anti‐IFN‐α receptor 1 antibody and two phase III clinical trials (TULIP‐1 and TULIP‐2) revealed that anifrolumab was well‐tolerated and effective in the improvement of skin symptoms.^46^ Humanized anti‐IFN‐α monoclonal antibodies such as sifalimumab are promising new treatment agents and future trials are expected.^46^

## CONFLICT OF INTEREST STATEMENT

None declared.
